# Impact of different stall layouts with robotic milking systems on the behavioral pattern of multiparous cows

**DOI:** 10.3168/jdsc.2023-0505

**Published:** 2024-04-20

**Authors:** Priscilla Ayleen Bustos Mac-Lean, Leonardo Alexander de Oliveira Campos, Camila Pires Cremasco, Eduardo Guilherme Satolo, Liliane Ubeda Morandi Rotoli, Odriom Escobar, Samuel Victor Brasilino Feitosa

**Affiliations:** 1 Department of Biosystems Engineering, São Paulo State University (Unesp), School of Sciences and Engineering, Campus Tupa 17602496, Brazil; 2 Department of Management, Development and Technology, São Paulo State University (Unesp), School of Sciences and Engineering, Campus Tupa 17602496, Brazil; 3 School of Philosophy and Sciences, São Paulo State University (Unesp), Campus Marília 17525900, Brazil; 4 Agrícola Ancalí Ltda., VIII Región, Chile; 5 Graduate Program in Agribusiness and Development, São Paulo State University (Unesp), School of Sciences and Engineering, Campus Tupa 17602496, Brazil

## Abstract

The present study aimed to compare the efficiency of different pens and animal flow configuration layouts in freestall pens using a robotic milking system (RMS) with guided flow based on the behavioral patterns of multiparous lactating Holstein dairy cows in a commercial farm. The behavior of 24 cows in freestall pens was evaluated, divided into 4 different stall configurations: original (OR), conversion (CVS), toll-booth I (TBI), and toll-booth II (TBII), each featuring distinct circulation layouts with different configurations of location, position, and number of guided-flow RMS equipment, feed bunk, water trough, commitment pen, sand beds, sorting gates, and one-way gate. Six multiparous cows, parity 2 or 3, with an average (± SD) of 180 ± 20 DIM, were randomly selected from each freestall pen for focal assessment of behavioral patterns. The location, position, and behavior of each animal were recorded in a field ethogram, with individual sequences recorded at 15-min intervals using the focal method during 6 nonconsecutive 10-h periods in each pen, and the proportion of observed time for each behavior was assessed. The pens differed in the number of available milking robots (1, 2, or 3), the number of animals per robot, the quantity and orientation of smart doors, and the placement of feed bunks, water troughs, and sand beds. A completely randomized experimental design was used to compare the 4 stalls, with a nonparametric Kruskal-Wallis test, in which the medians of the treatments were then compared with the Dunn test at a significance level of 5%, using the Minitab software. The behavioral pattern of cows exhibited differences based on the stall configuration with RMS as a proportion of the observed time (60 h). The TBI stall configuration, where the animal needs to be milked to exit the milking robot, showed a higher percentage of observed time spent in the sand bed (68%) and lying position (64%) compared with other stalls, as a proportion of the observed time (60 h). Notably, the TBII stall exhibited a significant amount as a proportion of observed time (60 h) in the holding area (16%), possibly attributed to a layout with a higher number of animals per robot, emphasizing the importance of respecting the number of animals per robot when housing in a stall with RMS. Thus, spatial configuration and the density of robotic systems are factors that influence the behavioral pattern of dairy cows.

## Highlights

• The efficiency of using a freestall depends on several factors.
• The animal density in a freestall affects the behavioral profile of dairy cows.
• The layout of stalls with RMS influences the behavior of multiparous dairy cows.
• The stress of the cow is reduced with the management strategies.
• The capacity rate of a stall determines the efficiency of a robotic milking system.

## Graphical Abstract

**Figure:**
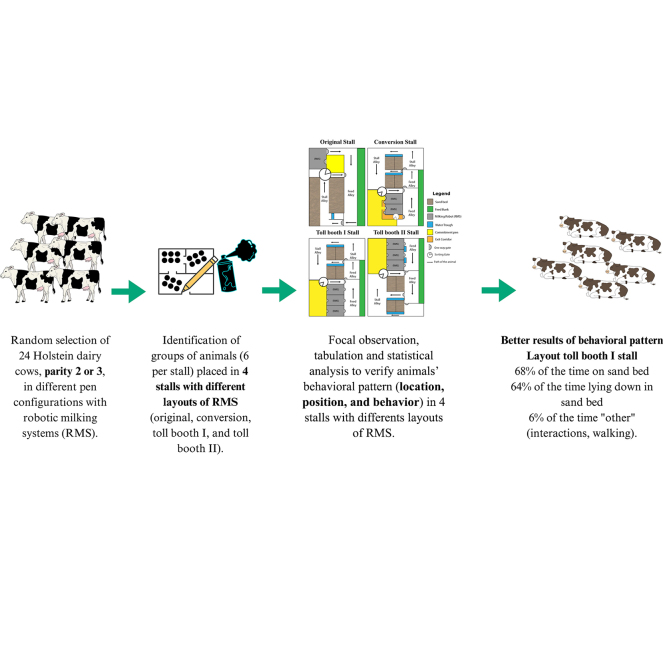
**Summary** This study compares the efficiency of different pens and animal flow configuration layouts in freestall pens using a robotic milking system. A sample of 24 Holstein dairy cows of parity order (2 or 3) was divided into groups of 4 different stall configurations: original, conversion, toll booth I, and toll booth II. Each type of layout had different physical configurations of location, position, and equipment. Analyses of animal localization, position, and behavior were performed, followed by statistical analysis. In the toll booth I pen, the animals spent 68% of their time in bed, 64% of the time sleeping, and 6% of the time engaged in “other” behaviors, such as walking and interacting, in 60 hours of observation. The spatial distribution provided by this layout indicates the highest level of well-being and the lowest incidence of undesirable side effects. Behaviors in confined groups, such as “walking” and “interacting,” are mostly negative.

Milk is a globally consumed food product; therefore, the efficient production of this product is of great importance to the world, given the increasing concern for the efficient use of natural resources versus food productivity (Bhat et al., 2022) and animal welfare considerations (John et al., 2016). Providing an appropriate environment for animals is crucial when aiming for productive efficiency (Dos Santos et al., 2021). Stress leads to changes in the behavior and physiology of animals, characterized by irritability, loss of appetite, and aggression, adversely affecting animal performance and health (Nicodemo et al., 2018). Simple human contact or changes in animal routines can induce stress levels, ranging from poor to very good welfare level, defined as an environmental stimulus (external to the animal caused by humans, climatic variables or installations) with the potential to overload or challenge the animal's homeostatic systems (Broom, 2011).

Milking moments for dairy cows are stressful, and the use of robotic milking systems (**RMS**) is a favorable option to reduce human intervention and disruption of the animal's routine.

Ji et al. (2022) stated that RMS use devices and sensors capable of collecting data on various factors such as environmental conditions, animal health, milk quality, and especially, productivity (Sitkowska et al., 2015). However, current literature lacks data on animal behavior within RMS. The cycle leading up to milking and the layout configuration of RMS vary according to the farms or the animals themselves. However, there is a lack of studies identifying key points to be observed or comparing different installation layouts. The objective of this study was to compare the efficiency of different installation layouts using RMS based on the behavioral pattern of multiparous lactating cows on a commercial farm. The study assessed the percentage (%) of time that animals spent in specific locations within the stall, as well as their position and behavior for 10 h per day, observed over 6 nonconsecutive days in each pen.

The project received approval from the Ethics Committee for Animal Use (CEUA) of the School of Sciences and Engineering (São Paulo State University), under process number 02/2023. The study followed all technical recommendations for animal studies presented by the committee, ensuring that there was no unnecessary discomfort to the animals through proper management or observation. The project was conducted between February and March 2023 on a commercial dairy farm located in southern Chile, milking around 5,000 Holstein cows daily, using DeLaval RMS.

Four freestall pens with different configurations of location, position, and number of guided-flow RMS equipment, feed bunk, water trough, commitment pen, sand beds, sorting gates, and one-way gate (Figure 1) were used in the study.

**Figure 1:**
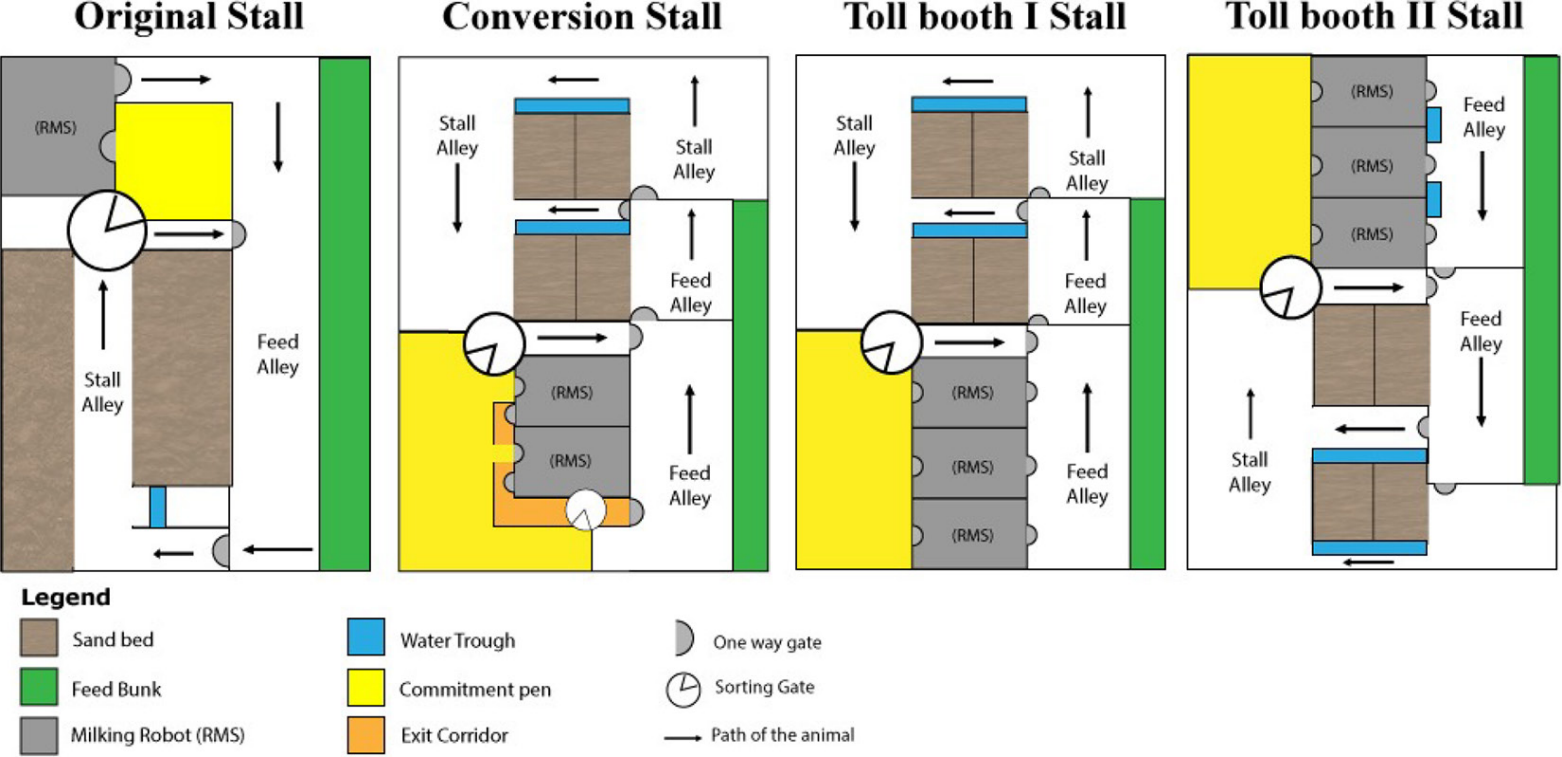
Freestalls with different circulation layouts for lactating cows, using RMS. The arrow indicates the direction in which the animals move. Source: the authors.

The stalls are described as follows: In the original (**OR**) layout stall, a total of 64 lactating cows are housed, including 27 multiparous cows of parity 2 or 3. There are 65 sand beds covered with sand in their resting area. The stall features one milking robot (65 animals/robot), located at one end of the stall. To undergo milking, animals must pass through a sorting gate, with entry to the commitment pen allowed only if at least 4 h have passed since the last milking. The animal enters the RMS when it is unoccupied. After milking, the animal is directed to the feed bunk sector through a one-way gate. However, if the time since the last milking is less than 4 h, the animal is directed to the feed bunk sector without passing through the holding area and milking robot. The system consists of a central stall alley serving as the main circulation route for cows. There is a holding area designated for cows entering the milking robot, followed by a feed alley providing access to the feed bunk and subsequently a one-way gate providing access to the stall alley with a water trough area. When a cow needs water or wants to lie down, she must return to the stall alley through a one-way gate, restarting the process. Milking occurs in the robot, with an average duration of 8 min, during which the animal receives concentrated feed (according to production).

The conversion (**CVS**) layout stall accommodates 126 lactating animals, including 63 multiparous cows of parity 2 or 3, and has 135 sand beds. Cows have the option to choose milking in 2 in-line operating milking robots (67 animals/robot) with guided flow. This installation stands out for housing a larger number of animals compared with OR and features 2 different sorting gates—one for entering the holding area and milking robots, and another for exiting the holding area, as the milking robot's exit is directed to an exit corridor within the holding area. The first sorting gate determines whether the animal will enter the holding area or be directed to the feed alley, limited by the time since the last milking (>4 h: directed to the holding area; <4 h: directed to the feed alley). The entrance and exit of the milking robot are on the same side since the RMS is arranged in line (one behind the other). When the animal exits the milking robot, she is directed to an exit corridor, leading back to one of the sorting gates, potentially delaying the entry of other animals or congesting the milking process. In this layout, milked and unmilked cows are not separated, and a previously milked cow may re-enter the milking robot, causing delays and forming a loop. If the cow has been milked, upon passing through the sorting gate, she has access to the feed alley and feed bunk. If the animal desires water or rest, she must pass through a one-way gate to access the stall alley, where she will find the sand bed and water trough.

In the toll-booth I (**TBI**) stall, there were 171 lactating cows, including 70 multiparous cows of parity 2 or 3, with 154 sand beds. This installation includes 3 milking robots (57 animals/robot). The difference from the previous stalls is that the milking robots are positioned parallel to each other, and to access the feed bunk, the cow must “pay a toll” by passing through the milking robot. The sorting gate directs the cow to the holding area if it is time to be milked (minimum 4 h since the last milking); otherwise, the cow is directed to the feed alley through a one-way gate. This configuration effectively separates already milked cows from those yet to undergo milking, preventing an animal from getting stuck in a loop. After the feed bunk area, cows wishing to lie down or drink water must pass through a one-way gate and head to the sand bed and water trough area. If the cow wishes to eat, she must pass through the sorting gate again, and if it is time for a new milking, the cow must “pay a toll” to access the feed bunk. This design provides notable fluidity, allowing simultaneous milking of multiple animals.

The toll-booth II (**TBII**) stall has a configuration like TBI but accommodates 196 animals, including 105 multiparous cows of parity 2 or 3. This installation also includes 3 milking robots (65 animals/robot). This stall differs by having a slightly smaller holding area than TBI (8 m^2^ less) and having a water trough at the exit of the milking robots, which are arranged parallel to each other. Additionally, the milking robot's software is more updated (DelPro-V300, DeLaval), resulting in a shorter milking time (approximately 6 min).

To record the behavioral pattern of cows, 6 randomly selected multiparous females in the middle of the lactation cycle (average of 180 DIM) were chosen from each stall's total multiparous cow group, totaling 24 Holstein animals with an average milk production of 45 L/d. All animals housed on the farm are healthy, having undergone veterinary evaluation, and show no mobility issues (locomotion score less than or equal to 2, according to Robinson and Juarez, 2003). The animals were visually identified by numbers sprayed on the right and left flanks to facilitate observation.

Behavioral recordings were performed with individual sequence registrations at 15-min intervals using the focal method (Bateson and Martin, 2021). The sequence was kept consistent to avoid altering the time between animals. Behavioral pattern evaluations were conducted during 6 nonconsecutive days, with an average duration of 10 h in each of the 4 freestall pens (with different layouts), always at the same time.

The behavioral pattern of the animals was recorded in an ethogram (field table), dividing the records into location (sand bed, stall alley, feed bunk, feed alley, water trough, holding area, milking robot-RMS), position (standing or lying), and behavior (idleness, ruminating, eating, sleeping, other behaviors such as drinking, walking, interactions, and grooming), as a proportion of observed time (60 h).

A completely randomized experimental design was used, and as a sample size validation, considering an ANOVA with 4 treatments (stalls) and 6 repetitions (nonconsecutive behavior records), a sufficient sample size was obtained for proper analysis. The treatments were analyzed using the nonparametric Kruskal-Wallis test, and their medians were compared with the Dunn test at a 5% significance level. In the results tables, the representative values for each treatment illustrate mean values, followed by a corresponding letter for median comparison.

The cows' behavioral profile showed differences based on the layout configuration of the RMS facilities. Table 1 descriptively presents the relationship between the behavioral profile (measured as a percentage of the total observation time) and the different layouts.

**Table 1:** Medians ± SD of the frequency in percentage (%) of the behavioral pattern of multiparous dairy cows in mid lactation housed in facilities with different stall layouts of RMS^1^

Ethogram and layout	OR	CVS	TBI	TBII
Location				
Sand bed	60 ± 28^ab^	59 ± 27^ab^	68 ± 26^a^	45 ± 28^b^
Stall alley	7 ± 10^a^	5 ± 8^a^	4 ± 7^a^	7 ± 11^a^
Feed bunk	13 ± 17^a^	11 ± 17^a^	14 ± 17^a^	18 ± 20^a^
Feed alley	4 ± 10^a^	1 ± 5^a^	3 ± 8^a^	2 ± 4^a^
Water trough	3 ± 9^b^	13 ± 5^a^	4 ± 7^b^	3 ± 4^b^
Water trough alley	2 ± 5^a^	3 ± 14^a^	2 ± 6^a^	1 ± 5^a^
Holding area	9 ± 14^ab^	7 ± 12^ab^	5 ± 15^b^	16 ± 24^a^
RMS	1 ± 3^a^	2 ± 4^a^	1 ± 2^a^	2 ± 4^a^
Position				
Standing	45 ± 27^a^	49 ± 28^a^	36 ± 27^a^	53 ± 29^a^
Lying down	55 ± 27^ab^	51 ± 28^ab^	64 ± 27^a^	42 ± 28^b^
Behavior				
Idleness	35 ± 14^a^	37 ± 15^a^	36 ± 16^a^	34 ± 21^a^
Ruminating	21 ± 19^a^	27 ± 18^a^	27 ± 20^a^	21 ± 15^a^
Eating	13 ± 17^a^	13 ± 18^a^	13 ± 17^a^	18 ± 10^a^
Sleeping	20 ± 19^a^	12 ± 9^a^	19 ± 20^a^	14 ± 16^a^
Other^2^	12 ± 12^a^	10 ± 6^ab^	6 ± 8^b^	7 ± 6^ab^

a,b Different superscripts in the same row indicate significant differences in medians by Dunn's test at a 5% significance level (*P* < 0.05).

1 OR = original stall; CVS = conversion stall; TBI = toll-booth I stall; TBII = toll-booth II stall.

2 Drinking, walking, interaction, and grooming behavior.

Categorizing the behavior of dairy cows by location, position, and behavior provided a detailed view of their activities in the freestall. Regarding location, statistically significant variables were sand bed, water trough, and holding area. It was observed that animals in the TBI layout spent a significant (*P* < 0.05) portion of their time (68%) in the sand bed, where they could rest and ruminate. This indicates a good level of well-being and comfort, as animals spending more time lying down are in good well-being (Fregonesi and Leaver, 2002, Halachmi, 2000; Drissler et al., 2005), considering the provision of environmental domain as presented by Mellor and Beausoleil (2015). Thus, the TBI layout showed (*P* < 0.05) greater comfort for the observed animals compared with the TBII layout, which may also be explained by the lower number of animals per robot, potentially making the milking process more efficient.

Cows spent 13% of their time in the water trough when in the CVS layout, a much higher percentage compared with OR, TBI, and TBII layouts, with 3%, 4%, and 3% of the proportion of observed time, respectively. The proximity of the water trough may be related to its proximity to the grooming brush visited by the animals and embedded in the “other” behavior category, or to stressful factors leading animals to drink more water (Broom, 2011).

The waiting flow for milking was analyzed in the location category. Waiting for the cow to enter the milking robot reflects negative aspects, leaving the animal standing without any benefit. It was observed that in the TBII layout, cows spent 16% of the proportion of observed time in the holding area. There was a frequency difference between the TBII layout compared with TBI, and similarity to the OR and CVS configurations. Physically, what differentiates the TBI and TBII layouts is the milking robot system that operates milking and the greater number of animals in the pen. Although the TBII milking robot is faster, there is a loss of efficiency and an increase in the frequency of undesirable behaviors (standing for a long time) when the batch is larger. The position category contributes to verifying the resting behavior. The increased observed time standing may indicate thermal discomfort and cows increase time spent standing when heat stressed (e.g., Cook et al., 2007). This allows for improved heat exchange (Spencer, 2011). It was identified that in the TBI layout, cows were in the lying position for 64% of the time, significantly more than TBII, which showed a frequency of 42% of the observed time.

In the behavior category, the time spent in idleness, ruminating, eating, sleeping, and other behaviors such as drinking, walking, interactions, and scratching was evaluated. For these variables, only the “other” behavior showed differences between layouts and periods (*P* < 0.05). Figure 2 shows the distribution of variables in this category.

**Figure 2:**
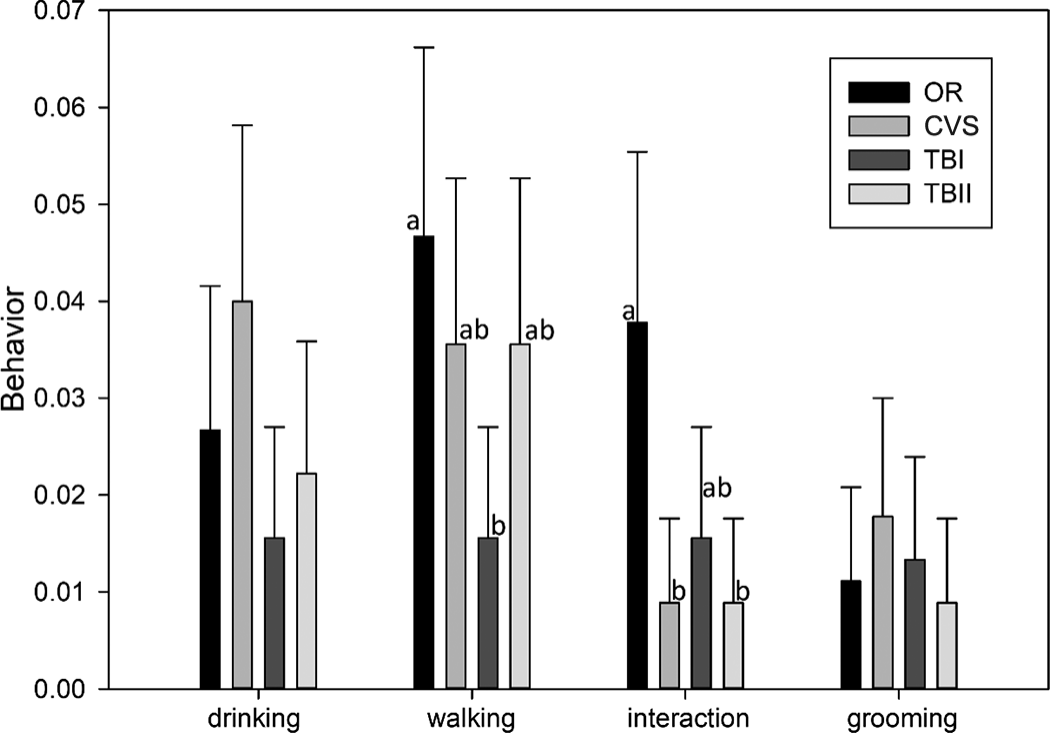
Breakdown of the time frequency (%) for the “other” behavior into drinking, walking, interaction, and grooming for different barns with original (OR), conversion (CVS), toll-booth I (TBI), and toll-booth II (TBII) layouts of multiparous dairy cows in mid lactation. Different lowercase letters on the same line indicate significant differences in medians by Dunn's test at a 5% significance level (*P* < 0.05). Error bars indicate SD.

In the “other” behavior, the statistically significant variables were “walking” and “interacting,” with the OR layout standing out. In terms of the frequency of time spent on “walking” behavior, the data indicated that the OR layout is higher compared with TBI and similar to CVS and TBII. Animal interaction is an important factor that indicates animal well-being, and the OR layout showed approximately twice the frequency of this behavior compared with other layouts. In the OR layout, only multiparous animals were housed, without the presence of primiparous ones, in a study presented by Morabito et al. (2017), it was found that the lactation stage and restricted rest time, especially in multiparous and more productive cows, pose a higher risk of aggressive behavior. Recurrent negative interaction behaviors included headbutting and pushing.

According to Halachimi (2000), the ideal layout model for robotic milking depends on the characteristics and objectives of each farm. The choice of the model should consider team management, work routine, cow behavior, feeding procedure, average waiting time of the cow, and local conditions. In this evaluation, the TBI layout showed better performance in relation to other configurations in terms of animal behavior, well-being, and spatial distribution within the freestall pen.

Thus, spatial configuration and the density of robotic systems are factors that influence the behavioral pattern of dairy cows.
